# Could the Calcium Silicate-Based Sealer Presentation Form Influence Dentinal Sealing? An In Vitro Confocal Laser Study on Tubular Penetration

**DOI:** 10.3390/ma14030659

**Published:** 2021-01-31

**Authors:** Paula Muedra, Leopoldo Forner, Adrián Lozano, José L. Sanz, Francisco J. Rodríguez-Lozano, Julia Guerrero-Gironés, Francesco Riccitiello, Gianrico Spagnuolo, Carmen Llena

**Affiliations:** 1 Department of Stomatology, Faculty of Medicine and Dentistry, Universitat de València, 46010 Valencia, Spain; P.muedra.bort@gmail.com (P.M.); adrianlozano@mac.com (A.L.); jsanzalex96@gmail.com (J.L.S.); llena@uv.es (C.L.); 2 Department of Dermatology, Stomatology, Radiology and Physical Medicine, Morales Meseguer Hospital, Faculty of Medicine, University of Murcia, 30100 Murcia, Spain; fcojavier@um.es (F.J.R.-L.); Julia.guerrero@um.es (J.G.-G.); 3 Department of Neurosciences, Reproductive and Odontostomatological Sciences, University of Naples “Federico II”, 80138 Napoli, Italy; riccitie@unina.it (F.R.); gspagnuo@unina.it (G.S.); 4 Institute of Dentistry, I. M. Sechenov First Moscow State Medical University, 119146 Moscow, Russia

**Keywords:** silicate-based sealers, dentinal tubule penetration, root canal sealing, presentation form, endodontics

## Abstract

Dentinal tubule penetration influences root canal treatment sealing. The aim of this study was to compare dentinal penetration of two clinical presentations of silicate-based sealers using confocal laser. Sixty single-rooted human teeth from 50–70 year-old patients extracted for orthodontic/periodontal reasons were used. Canals were prepared using Mtwo system up to 35/0.04, with 5.25% NaOCl irrigation and final irrigation using 17% EDTA. Teeth were randomly assigned into study groups (n_i_ = 20): EndoSequence BC sealer (ES, group 1), BioRoot RCS (BR, group 2); and a control group (n_c_ = 20) with AH Plus (AHP). Root canals were obturated with 35/0.04 gutta-percha (single-cone technique). The samples were obtained from apical, middle, and coronal thirds. Dentinal tubule penetration depth and percentage of penetration around the canal perimeter were measured. The statistical analysis was performed using Mann Whitney U test and Wilcoxon *t*-test (95% confidence interval). ES exhibited a significantly higher penetration than AHP in apical and middle thirds (*p* < 0.05), and in middle and coronal thirds relative to BR (*p* < 0.05). The percentage of penetration around the canal perimeter was significantly higher for ES compared to BR in all thirds, but only in the apical third for AHP (*p* < 0.05). The pre-mixed silicate-based sealer exhibited better penetration than the powder/liquid one.

## 1. Introduction

The objective of root canal obturation is to hermetically seal the canal system so that it is isolated, and thus prevent any leakage into or out of the root. The sealer applied in the process of obturation is responsible for dentin sealing, regardless of the technique used [1,2]. Different endodontic sealers have been developed for this purpose. Resin-based sealers have been the most widely used due to their low solubility, apical sealing ability, and handling [1]. However, their biocompatibility with periapical tissues is still a subject of study. Biocompatibility acts as a desirable biological property of endodontic sealers since, during root canal treatment, part of the biomaterial may inadvertently extrude from the root canal towards the surrounding tissues. This may result in an inflammatory response from such tissues, which may hinder the healing of any pre-existing lesions [3,4].

Under the present criteria, endodontic sealers are expected not only to seal the root canal system, but also promote the healing of injured periapical tissues to produce a biological apical seal.

Currently, new silicate-based sealers with a composition inspired by mineral trioxide aggregate (MTA) are available. These materials are formed, mainly, by calcium silicates and phosphates, and calcium hydroxide [5]. They are presented in two forms: Pre-mixed or in powder/liquid format.

EndoSequence BC Sealer (ES; Brasseler USA, Savannah, GA, USA) is a premixed silicate-based material presented in a ready-to-use format [6]. BioRoot RCS (BR; Septodont, Saint-Maur-des-Fossés, France) is also a silicate-based sealer, but it is presented in powder/liquid form, requiring it to be mixed prior to its use [7].

Several studies have verified the excellent biocompatibility [8,9] and bioactivity [4,10,11], as well as the antibacterial effect [12,13,14] of both sealers. Most importantly, by presenting bioactive properties, endodontic sealers are capable of forming a superficial layer of a mineralized tissue which favors the formation of a mineral attachment with the dentinal tissue [15]. Furthermore, they present an adequate fluidity [4,5,6] and dimensional stability [16], which directly influence the quality of the canal obturation.

Dentine tubular penetration represents a physical barrier for microorganisms and increases the retention of the sealer [17,18], hence, the importance of its assessment in these new materials. Among the factors that determine the tubular penetration of root canal sealers, the consistency and fluidity are two relevant parameters to be considered.

Confocal Laser Scanning Microscopy (CLSM) is a widely used technique for the study of tubular penetration of various materials. Several published studies in the field use this method to evaluate the penetration of sealing cements [16,17,18,19]. However, there is limited evidence regarding the difference of dentinal tubule penetration between premixed and powder/liquid silicate-based sealers specifically [19].

Due to the increasing interest and clinical use of these endodontic biomaterials, there is a need to expand the existent information in this regard. The identification of significant differences in tubular penetration between the different presentation forms of the sealers could potentially lead to an increased clinical use of one form or another.

Accordingly, the objective of the present in vitro study was to evaluate the dentinal tubular penetration of different endodontic sealers by comparing two silicate-based sealers, one premixed and the other in powder/liquid form with each other, and with a resin-based sealer; by means of an analysis with confocal laser scanning microscopy. The null hypothesis was that there is no difference between the tested materials in their tubular penetration.

## 2. Materials and Methods

### 2.1. Sample Preparation

Maxillary and mandibular single-rooted teeth extracted from 50–70 year-old patients due to periodontal or orthodontic reasons were selected (N = 60), all of which presented a type I canal configuration according to Weine’s classification [20], and an apical curvature of less than 5° [21] following Schneider’s technique [22]. The present in vitro study was approved by the ethics committee from the *Universitat de València* (ref. H1460624886119). Teeth with root resorptions, carious lesions (coronal or root lesions), immature apices, fractures, or previous root canal treatments were excluded.

The selected teeth were decoronated at 15 mm from the apex to standardize sample lengths and they were prepared at a working length of 14 mm with the Mtwo rotary system (VDW, Munich, Germany) up to a 35/0.04 file, having previously achieved patency using K10 files (Dentsply Sirona, York, PA, USA). Irrigation during instrumentation was performed using 3 mL of 5.25% sodium hypochlorite (Dentaflux, Madrid, Spain), and final irrigation was carried out with 17% EDTA (DIRECTA AB, Stockholm, Sweden) during 1 min, followed by 5 mL saline solution.

Samples were randomly assigned to 2 study groups (n_i_ = 20), according to the silicate-based sealers: group 1, EndoSequence BC Sealer (ES); group 2, BioRoot RCS (BR), and a control group (n_c_ = 20) AH Plus (AHP). The sample size was established by comparison with similar studies in the field [19,23]. The composition, manufacturers, and batch numbers of each sealer, are included in Table 1.

Endodontic sealers were prepared following their manufacturers’ instructions, adding a fluorochrome (0.1% Rhodamine B, C_28_H_31_ClN_2_O_3_, Panreac Químicas S.A.U. Casteller del Vallès, BCN, Spain) to make them visible under confocal laser scanning microscopy (CLSM). One g of endodontic sealer was weighed on an analytic scale (Shimadzu, Tokyo, Japan) with a precision of 0.0001 g. Next, 0.002 g of the Rhodamine B was weighed.

Once prepared, the materials were inserted into the root canals with the aid of a 25 caliber lentulo spiral filler at 150 rpm, 1 mm from the apex. The root canals were then obturated with 35/0.04 gutta-percha (FKG Dentaire S.A., La Chaux-de-Fonds, Switzerland) by means of the single-cone technique.

During the storage period, before and after the instrumentation and obturation, the roots were kept in a 100% humidity environment. After a minimum of 72 h of storage, to ensure the complete setting of the materials, samples were obtained from the apical, middle, and coronal thirds at 3, 5, and 8 mm from the root apex, respectively, using a diamond disc to obtain a 1 mm section of each third. The obtained samples were fixed onto microscope slides and were polished using fine and superfine grain SoftLex discs (3 M ESPE, St. Paul, MN, USA). The samples were then stored in a light-free environment to avoid a previous exposure of the fluorochrome to light before it was viewed under CLSM.

### 2.2. CLSM Analysis

**Figure 1:**
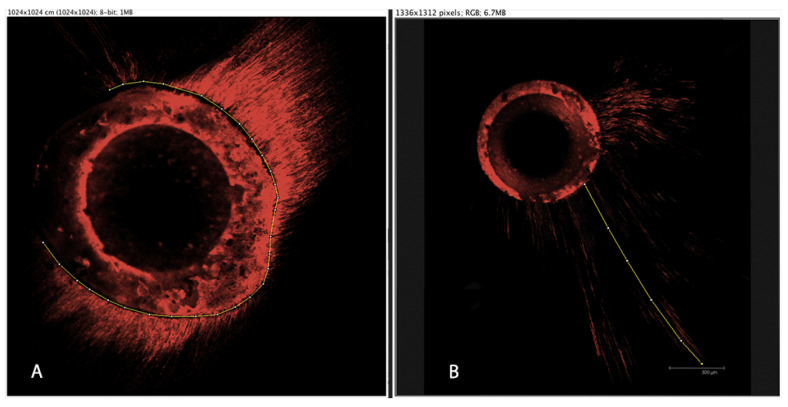
Representative images of the measurement of the tubular perimeter penetrated by cement (**A**) and the dentin tubule penetration depth (panel **B**). The central rounded unstained portion of the image represents gutta-percha. The surrounding stained portion represents the sealer. The prolongations of the stained portion represent the sealer penetration inside the dentinal tubules.

The equipment used for the microscopic study was CLSM Leica TCS SP2 (Leica Microsystems GmbH, Wetzlar, Germany) with 543 nm and 578 nm excitation and emission wavelengths, respectively. Sample analysis was performed using 10× magnification. Images were registered and posteriorly analyzed via ImageJ v1.51 software (National Institutes of Health, Bethesda, MD, USA). Dentinal tubule penetration depth (in μm) and the percentage of penetration around the canal perimeter was measured from the images obtained by CLSM (Figure 1).

### 2.3. Data Analysis

Data obtained were statistically analyzed using IBM^®^ SPSS V25.00 (IBM^®^ SPSS Statistics, Inc., Chicago, IL, USA). Distribution was evaluated by the Kolmogorov Smirnov test, and it was determined that the variables did not present a normal distribution. Accordingly, values of penetration into dentinal tubules of the two silicate-based sealers were compared between them and with AH Plus sealer (control) in each root third (apical/middle/coronal) by U Mann Whitney test. Comparisons between thirds in each group was performed two by two by means of the Wilcoxon *T*-test. The confidence interval was established at 95%.

## 3. Results

The quantitative results of the CLSM image analysis in terms of the tubular penetration depth and percentage of dentinal tubule perimeter penetration are presented in Table 2. ES exhibited a significantly higher penetration than AHP (control) in the apical and middle thirds (*p* < 0.05), and in the middle and coronal thirds relative to BR. The percentage of penetration around the canal perimeter was significantly higher for ES compared to BR in all thirds, but only in the apical third for AHP (*p* < 0.05).

Comparisons within each group, between thirds were performed two by two by means of the Wilcoxon *T*-Test. Both in the silicate-based sealers and AHP groups, the penetration depth was significantly decreased from the coronal to the apical thirds (*p* < 0.05). In relation to the percentage of dentinal tubule perimeter penetrated by cement, ES group showed significant differences between the coronal and apical thirds (*p* < 0.01); BR group between the coronal and apical thirds, and between the coronal and middle thirds (*p* < 0.005); while AHP exhibited significant differences between all thirds (*p* < 0.05).

**Figure 2:**
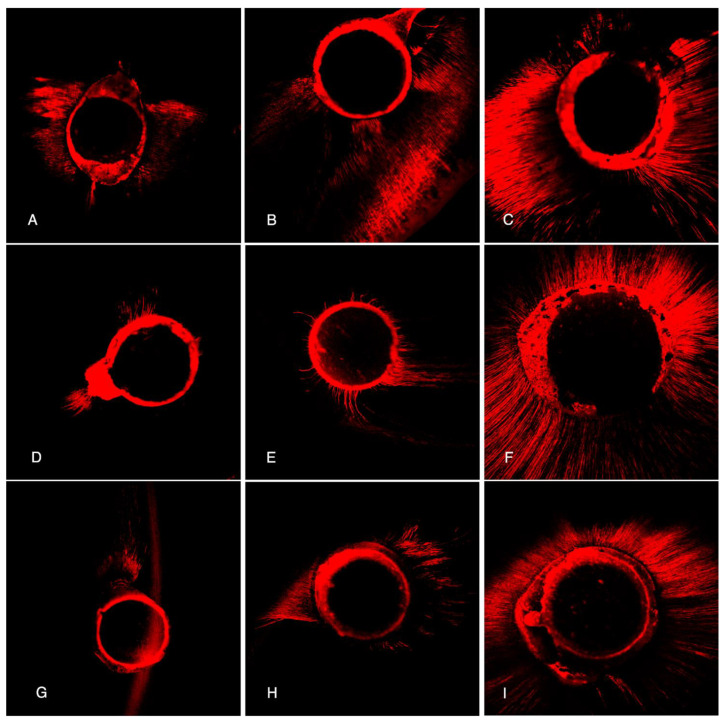
Representative CLSM images of each third for each of the studied sealers (10× magnification). EndosSequence: apical (**A**), middle (**B**), and coronal (**C**) thirds; AH Plus: apical (**D**), middle (**E**), and coronal (**F**) thirds; BioRoot: apical (**G**), middle (**H**), and coronal (**I**) thirds.

**Figure 3:**
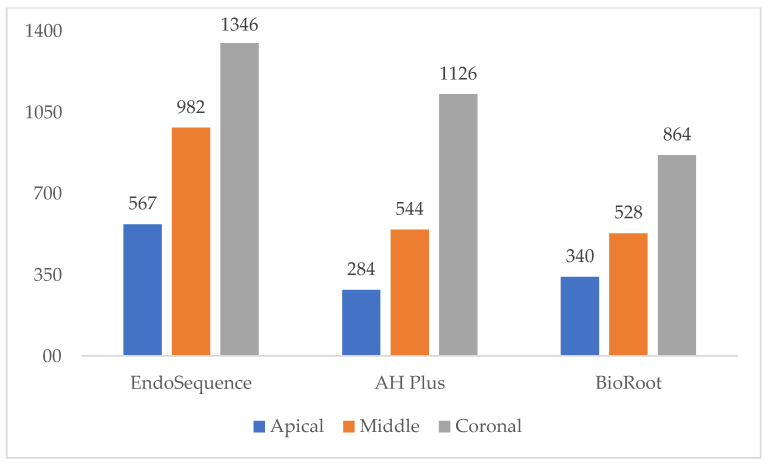
Mean tubular penetration depth by thirds.

Figure 2 illustrates images of the penetration depth and percentage of dentinal tubule perimeter penetration by sealers and by thirds. Complementarily, the differences in the mean tubular penetration depths between the groups are illustrated in Figure 3. In all thirds, penetration depth was significantly different between groups (*p* < 0.05).

## 4. Discussion

To the authors’ knowledge, the novelty of this paper resides in the fact that this is the first study to explore the differences in dentinal tubule penetration between silicate-based endodontic sealers in terms of their form of presentation (pre-mixed or powder/liquid).

ES and BR are two pure silicate-based sealers. Although they present a similar composition, they differ in their presentation. While ES comes premixed, in a ready-to-use format, BR is available in powder/liquid form. This difference means that, despite the fact that both materials require water to start and complete their setting reaction, ES will only depend on the remaining humidity in the dentinal tubules, while BR starts its reaction when mixing it [24].

Currently, no endodontic sealer meets all the ideal properties expected of them, such as dimensional stability, biocompatibility, and adhesion to root canal walls [25]. However, AHP has established itself as the reference sealer by which to compare new materials that are introduced onto the market, as a result of its satisfactory properties [1].

CLSM is a widely used procedure for the study of tubular penetration [17,19,23,24,26,27,28], as it allows to observe the penetration of the sealer throughout the dentinal tubule. This is possible since the microscope makes various cuts of the sample at depths of 20–30 μm [29], and it superimposes them, providing a three-dimensional image that allows to illustrate the path the sealer has travelled inside the dentinal tubules.

Techniques like scanning electron microscopy (SEM) have also been used for the study of tubular penetration [30,31,32,33]. However, it does not grant a full assessment of the relationship between the sealer and dentine. This type of microscopy only studies the surface of the samples and not their whole extension due to the sinuous configuration of the dentinal tubules [29], thereby, providing a limited information compared to CLSM.

In relation to the results of the present study, ES showed a higher penetration compared to the other sealers although not significantly in all cases. This may be due, partially, to the size of the sealer particles (<1 μm) [34], which is considerably smaller than that of BR (5–30 μm) [35] and AHP (1.5–8 μm) [36]. In addition to its fluidity, this allows the sealer to reach greater depths within the dentinal tubules, with diameters between 2–3 μm [37].

El Hachem et al. [38] and Eymirli et al. [39] obtained greater penetration depths than in the present study for premixed bioceramic sealers (2060 versus 1316.57 (coronal) −475.35 μm (apical), respectively). Despite this, the trend, in terms of the distribution by thirds and the nature of the sealers, is consistent with our results, reporting a deeper penetration from ES than from AHP.

The median values showed by AHP are similar to those from various studies [26,40,41]. Although, Türker et al. [17] found higher penetration depths. This difference may be due to the fact that only samples from the root’s middle third were used, in contrast with the present study. On the other hand, Singh et al. [31] and Shokouhinejad et al. [30] obtained penetration depth values which were noticeably inferior to our results (21 versus 24 μm, respectively). This difference may be explained by the microscopic technique used in both studies (SEM) and the limitations it presents for the study of tubular penetration.

The assessment of the percentage of penetration around the canal perimeter has an important clinical relevance, acting as a representation of the sealing ability against microorganisms in the dentinal tubules, regardless of the depth of penetration of the sealer [33,42]. Therefore, this parameter was included in our study.

Wang et al. [41] reported the same methodology and sealers as in the present study, and obtained similar percentage values. On the other hand, McMichael et al. [42] also found lower percentage values in resin-based materials when compared to silicate-based materials using single-cone technique, but the results for resin-based materials were superior when using continuous wave technique. The results from the study performed by Kim et al. [18] are also consistent with these findings. In the present study, the single-cone technique was used as a standardized obturation technique for all of the sealer samples, aiming to avoid the influence of the differences in obturation technique on the results. However, as described previously, this technique is not the most adequate when used with resin-based sealers such as AHP [42]. Therefore, the results obtained from this group may have been undervalued.

Despite the fact that the tubular penetration of BR was not significantly different from that of AHP, the values obtained in our study were lower than ES. The difference in penetration between both silicate-based sealers may be due to their different forms of presentation. Even having followed the manufacturers’ instructions for the preparation of BR, small variations in the dosage during the mixing process may have been produced. Consequently, this may have acted as a limitation, potentially having an effect on the final results by varying the fluidity of the sealer, and thereby, influencing its tubular penetration. Since ES is presented in a premixed, ready-to-use format, its fluidity and consistency are not altered, so they are always optimal.

Despite using human teeth extracted for different reasons (orthodontic or periodontal), the tubular penetration pattern followed the same trend in all teeth. Both for the depth of penetration inside the dentinal tubule and for the percentage of tubular penetration, the apical third showed the lowest results, followed by the middle and coronal thirds; coinciding with other authors [17,24,31,36,41,42]. This may be attributed to the differences in dentinal tubule distribution and caliber depending on the root third. The number and size of dentinal tubules decreases in a corono-apical direction. Similarly, a higher number of sclerosed tubules can be found in the apical third [43]. In addition, the difficulty of irrigant solutions to reach the apical areas of the root canal system hinders the removal of the smear layer generated by the instrumentation, preventing the entrance of the sealer due to tubule obliteration [44,45]. Consequently, regardless of the obturation technique and the nature of the sealer, the tendency is for tubular penetration to increase apico-coronally [46,47].

The ready-to-use attribute from the premixed silicate-based sealers, together with their potentially higher dentinal tubule sealing exhibited in the present study, could act as supporting evidence for their clinical use versus powder/liquid sealers. However, the lack of similar studies regarding this comparison can act as a limitation for the applicability of the results of the present study. In order to extrapolate the results into the clinical setting, future studies comparing similar biomaterials under different conditions should be performed.

ES exhibited the highest tubular penetration, while no significant differences were found between BR and AHP. Both the maximum depth of penetration inside the dentinal tubules and the percentage of penetration presented higher values in the coronal thirds for all the studied materials. Within the limitations of the present study, it can be concluded that tubular penetration was higher in the premixed endodontic sealer.

## Figures and Tables

**Table 1:** Composition of the materials used in the study.

Root Canal Sealer	Composition	Manufacturer
EndoSequence BC Sealer (ES) Lot. 12002SP	Zirconium oxide, calcium silicates, calcium phosphate monobasic, calcium hydroxide, filler, thickening agents	Brasseler USA, Savannah, GA, USA.
BioRoot RCS (BR) Lot. B20657	Powder: tricalcium silicate, zirconium oxide, povidone; Liquid: aqueous solution of calcium chloride, polycarboxylate	Septodont, Saint-Maur-des-Fossés, France
AH Plus (AHP) Lot. 1407000884	Paste A: bisphenol A epoxy resin, bisphenol F epoxy resin, calcium tungstate, zirconium oxide, silica, iron oxide pigments; Paste B: dibenzylamine, aminoadamantane, tricyclodecane-diamide, calcium tungstate, zirconium oxide, silica, silicone oil	Dentsply Sirona, York, PA, USA.

**Table 2:** Cement penetration depth inside dentinal tubules, divided by thirds and cements.

	Tubular Penetration Depth						Percentage of Perimeter Penetrated					
*n* = 20 in Each Group and Third	Apical		Middle		Coronal		Apical		Middle		Coronal	
	Median	IQR	Median	IQR	Median	IQR	Median	IQR	Median	IQR	Median	IQR
EndoSequence BC Sealer (ES)	475.35 ^a^	404.81	981.33 ^a,b^	581.14	1316.57 ^a^	804.57	33.65 ^a,b^	52.15	49.73 ^a^	44.23	55.64 ^a^	43.23
AH Plus (AHP)	193.25 ^a^	431.55	449.03 ^a^	612.64	1118.01	727.53	11.8 ^a^	13.25	39.67	28.83	60.37	27.14
BioRoot RCS (BR)	304.32	505.02	508.11 ^b^	715.84	743.74 ^a^	781.18	9.76 ^b^	29.75	24.08 ^a^	41.7	28.41 ^a^	34.98

Median and interquartile range (IQR). The same letters in superscript indicate significant differences between groups by column. (U Mann Whitney test).

## Data Availability

The data presented in this study are available on request from the corresponding author.

## References

[1] Marciano M.A., Guimaraes B.M., Ordinola-Zapata R., Bramante C.M., Cavenago B.C., Garcia R.B., Bernardineli N., Andrade F.B., Moraes I.G., Duarte M.A. (2011). Physical properties and interfacial adaptation of three epoxy resin-based sealers. J. Endod..

[2] Moinzadeh A.T., Zerbst W., Boutsioukis C., Shemesh H., Zaslansky P. (2015). Porosity distribution in root canals filled with gutta percha and calcium silicate cement. Dent. Mater..

[3] Huang T.H., Yang J.J., Li H., Kao C.T. (2002). The biocompatibility evaluation of epoxy resin-based root canal sealers in vitro. Biomaterials.

[4] Zordan-Bronzel C.L., Tanomaru-Filho M., Rodrigues E.M., Chavez-Andrade G.M., Faria G., Guerreiro-Tanomaru J.M. (2019). Cytocompatibility, bioactive potential and antimicrobial activity of an experimental calcium silicate-based endodontic sealer. Int. Endod. J..

[5] Al-Haddad A., Che Ab Aziz Z.A. (2016). Bioceramic-Based Root Canal Sealers: A Review. Int. J. Biomater..

[6] Lee J.K., Kwak S.W., Ha J.H., Lee W., Kim H.C. (2017). Physicochemical Properties of Epoxy Resin-Based and Bioceramic-Based Root Canal Sealers. Bioinorg. Chem. Appl..

[7] Siboni F., Taddei P., Zamparini F., Prati C., Gandolfi M.G. (2017). Properties of BioRoot RCS, a tricalcium silicate endodontic sealer modified with povidone and polycarboxylate. Int. Endod. J..

[8] Santos J.M., Coelho C.M., Sequeira D.B., Marques J.A., Pereira J.F., Sousa V., Palma P.J., Santos A.C. (2021). Subcutaneous Implantation Assessment of New Calcium-Silicate Based Sealer for Warm Obturation. Biomedicines.

[9] Pedano M.S., Li X., Camargo B., Hauben E., De Vleeschauwer S., Yoshihara K., Van Landuyt K., Yoshida Y., Van Meerbeek B. (2020). Injectable phosphopullulan-functionalized calcium-silicate cement for pulp-tissue engineering: An in-vivo and ex-vivo study. Dent. Mater..

[10] Collado-Gonzalez M., Garcia-Bernal D., Onate-Sanchez R.E., Ortolani-Seltenerich P.S., Lozano A., Forner L., Llena C., Rodriguez-Lozano F.J. (2017). Biocompatibility of three new calcium silicate-based endodontic sealers on human periodontal ligament stem cells. Int. Endod. J..

[11] Jafari F., Jafari S., Etesamnia P. (2017). Genotoxicity, Bioactivity and Clinical Properties of Calcium Silicate Based Sealers: A Literature Review. Iran. Endod. J..

[12] Candeiro G.T., Moura-Netto C., D’Almeida-Couto R.S., Azambuja-Júnior N., Marques M.M., Cai S., Gavini G. (2016). Cytotoxicity, genotoxicity and antibacterial effectiveness of a bioceramic endodontic sealer. Int. Endod. J..

[13] Singh G., Elshamy F.M., Homeida H.E., Boreak N., Gupta I. (2016). An in vitro Comparison of Antimicrobial Activity of Three Endodontic Sealers with Different Composition. J. Contemp. Dent. Pract..

[14] Poggio C., Riva P., Chiesa M., Colombo M., Pietrocola G. (2017). Comparative cytotoxicity evaluation of eight root canal sealers. J. Clin. Exp. Dent..

[15] Tanomaru-Filho M., Torres F.F.E., Chavez-Andrade G.M., de Almeida M., Navarro L.G., Steier L., Guerreiro-Tanomaru J.M. (2017). Physicochemical Properties and Volumetric Change of Silicone/Bioactive Glass and Calcium Silicate-based Endodontic Sealers. J. Endod..

[16] Ordinola-Zapata R., Bramante C.M., Graeff M.S., del Carpio Perochena A., Vivan R.R., Camargo E.J., Garcia R.B., Bernardineli N., Gutmann J.L., de Moraes I.G. (2009). Depth and percentage of penetration of endodontic sealers into dentinal tubules after root canal obturation using a lateral compaction technique: A confocal laser scanning microscopy study. Oral Surg. Oral Med. Oral Pathol. Oral Radiol. Endod..

[17] Aktemur Turker S., Uzunoglu E., Purali N. (2018). Evaluation of dentinal tubule penetration depth and push-out bond strength of AH 26, BioRoot RCS, and MTA Plus root canal sealers in presence or absence of smear layer. J. Dent. Res. Dent. Clin. Dent. Prospect..

[18] Kim Y., Kim B.S., Kim Y.M., Lee D., Kim S.Y. (2019). The Penetration Ability of Calcium Silicate Root Canal Sealers into Dentinal Tubules Compared to Conventional Resin-Based Sealer: A Confocal Laser Scanning Microscopy Study. Materials.

[19] Weine F.S., Healey H.J., Gerstein H., Evanson L. (1969). Canal configuration in the mesiobuccal root of the maxillary first molar and its endodontic significance. Oral Surg. Oral Med. Oral Pathol..

[20] Seidberg B.H., Altman M., Guttuso J., Suson M. (1973). Frequency of two mesiobuccal root canals in maxillary permanent first molars. J. Am. Dent. Assoc..

[21] Schneider S.W. (1971). A comparison of canal preparations in straight and curved root canals. Oral Surg. Oral Med. Oral Pathol..

[22] Kebudi Benezra M., Schembri Wismayer P., Camilleri J. (2018). Interfacial Characteristics and Cytocompatibility of Hydraulic Sealer Cements. J. Endod..

[23] Grossman L.I. (1958). An improved root canal cement. J. Am. Dent. Assoc..

[24] Chandra S.S., Shankar P., Indira R. (2012). Depth of penetration of four resin sealers into radicular dentinal tubules: A confocal microscopic study. J. Endod..

[25] Arikatla S.K., Chalasani U., Mandava J., Yelisela R.K. (2018). Interfacial adaptation and penetration depth of bioceramic endodontic sealers. J. Conserv. Dent..

[26] El Hachem R., Le Brun G., Le Jeune B., Pellen F., Khalil I., Abboud M. (2018). Influence of the EndoActivator Irrigation System on Dentinal Tubule Penetration of a Novel Tricalcium Silicate-Based Sealer. Dent. J..

[27] Tedesco M., Chain M.C., Bortoluzzi E.A., da Fonseca Roberti Garcia L., Alves A.M.H., Teixeira C.S. (2018). Comparison of two observational methods, scanning electron and confocal laser scanning microscopies, in the adhesive interface analysis of endodontic sealers to root dentine. Clin. Oral Investig..

[28] Mamootil K., Messer H.H. (2007). Penetration of dentinal tubules by endodontic sealer cements in extracted teeth and in vivo. Int. Endod. J..

[29] Balguerie E., van der Sluis L., Vallaeys K., Gurgel-Georgelin M., Diemer F. (2011). Sealer penetration and adaptation in the dentinal tubules: A scanning electron microscopic study. J. Endod..

[30] Shokouhinejad N., Sabeti M., Gorjestani H., Saghiri M.A., Lotfi M., Hoseini A. (2011). Penetration of Epiphany, Epiphany self-etch, and AH Plus into dentinal tubules: A scanning electron microscopy study. J. Endod..

[31] Singh C.V., Rao S.A., Chandrashekar V. (2012). An in vitro comparison of penetration depth of two root canal sealers: An SEM study. J. Conserv. Dent..

[32] Soheilipour E., Kheirieh S., Madani M., Akbarzadeh Baghban A., Asgary S. (2009). Particle size of a new endodontic cement compared to Root MTA and calcium hydroxide. Iran. Endod. J..

[33] Reszka P., Nowicka A., Lipski M., Dura W., Drozdzik A., Wozniak K. (2016). A Comparative Chemical Study of Calcium Silicate-Containing and Epoxy Resin-Based Root Canal Sealers. Biomed. Res. Int..

[34] Chang S.W., Lee Y.K., Zhu Q., Shon W.J., Lee W.C., Kum K.Y., Baek S.H., Lee I.B., Lim B.S., Bae K.S. (2015). Comparison of the rheological properties of four root canal sealers. Int. J. Oral Sci..

[35] Cleghorn B.M., Goodacre C.J., Christie W.H. (2008). Morphology of teeth and their root canal systems. Endodontics.

[36] El Hachem R., Khalil I., Le Brun G., Pellen F., Le Jeune B., Daou M., El Osta N., Naaman A., Abboud M. (2019). Dentinal tubule penetration of AH Plus, BC Sealer and a novel tricalcium silicate sealer: A confocal laser scanning microscopy study. Clin. Oral Investig..

[37] Eymirli A., Sungur D.D., Uyanik O., Purali N., Nagas E., Cehreli Z.C. (2019). Dentinal Tubule Penetration and Retreatability of a Calcium Silicate-based Sealer Tested in Bulk or with Different Main Core Material. J. Endod..

[38] Piai G.G., Duarte M.A.H., Nascimento A.L.D., Rosa R.A.D., So M.V.R., Vivan R.R. (2018). Penetrability of a new endodontic sealer: A confocal laser scanning microscopy evaluation. Microsc. Res. Tech..

[39] Russell A., Friedlander L., Chandler N. (2018). Sealer penetration and adaptation in root canals with the butterfly effect. Aust. Endod. J..

[40] Jeong J.W., DeGraft-Johnson A., Dorn S.O., Di Fiore P.M. (2017). Dentinal Tubule Penetration of a Calcium Silicate-based Root Canal Sealer with Different Obturation Methods. J. Endod..

[41] Wang Y., Liu S., Dong Y. (2018). In vitro study of dentinal tubule penetration and filling quality of bioceramic sealer. PLoS ONE.

[42] McMichael G.E., Primus C.M., Opperman L.A. (2016). Dentinal Tubule Penetration of Tricalcium Silicate Sealers. J. Endod..

[43] Silva R.V., Silveira F.F., Horta M.C., Duarte M.A., Cavenago B.C., Morais I.G., Nunes E. (2015). Filling Effectiveness and Dentinal Penetration of Endodontic Sealers: A Stereo and Confocal Laser Scanning Microscopy Study. Braz. Dent. J..

[44] Moon Y.M., Shon W.J., Baek S.H., Bae K.S., Kum K.Y., Lee W. (2010). Effect of final irrigation regimen on sealer penetration in curved root canals. J. Endod..

[45] Aydin Z.U., Ozyurek T., Keskin B., Baran T. (2019). Effect of chitosan nanoparticle, QMix, and EDTA on TotalFill BC sealers’ dentinal tubule penetration: A confocal laser scanning microscopy study. Odontology.

[46] Akcay M., Arslan H., Durmus N., Mese M., Capar I.D. (2016). Dentinal tubule penetration of AH Plus, iRoot SP, MTA fillapex, and guttaflow bioseal root canal sealers after different final irrigation procedures: A confocal microscopic study. Lasers Surg. Med..

[47] Macedo L.M.D., Silva-Sousa Y., Silva S., Baratto S.S.P., Baratto-Filho F., Abi Rached-Júnior F.J., Jacob F. (2017). Influence of Root Canal Filling Techniques on Sealer Penetration and Bond Strength to Dentin. Braz. Dent. J..

